# Chemotaxis and Binding of *Pseudomonas aeruginosa* to Scratch-Wounded Human Cystic Fibrosis Airway Epithelial Cells

**DOI:** 10.1371/journal.pone.0150109

**Published:** 2016-03-31

**Authors:** Christian Schwarzer, Horst Fischer, Terry E. Machen

**Affiliations:** 1 Department of Molecular and Cell Biology, University of California, Berkeley, California, United States of America; 2 Children’s Hospital Oakland Research Institute, Oakland, California, United States of America; University of Alabama at Birmingham, UNITED STATES

## Abstract

Confocal imaging was used to characterize interactions of *Pseudomonas aeruginosa* (PA, expressing GFP or labeled with Syto 11) with CF airway epithelial cells (CFBE41o-, grown as confluent monolayers with unknown polarity on coverglasses) in control conditions and following scratch wounding. Epithelia and PAO1-GFP or PAK-GFP (2 MOI) were incubated with Ringer containing typical extracellular salts, pH and glucose and propidium iodide (PI, to identify dead cells). PAO1 and PAK swam randomly over and did not bind to nonwounded CFBE41o- cells. PA migrated rapidly (began within 20 sec, maximum by 5 mins) and massively (10–80 fold increase, termed “swarming”), but transiently (random swimming after 15 mins), to wounds, particularly near cells that took up PI. Some PA remained immobilized on cells near the wound. PA swam randomly over intact CFBE41o- monolayers and wounded monolayers that had been incubated with medium for 1 hr. Expression of CFTR and altered pH of the media did not affect PA interactions with CFBE41o- wounds. In contrast, PAO1 swarming and immobilization along wounds was abolished in PAO1 (PAO1ΔcheYZABW, no expression of chemotaxis regulatory components *cheY*, *cheZ*, *cheA*, *cheB* and *cheW*) and greatly reduced in PAO1 that did not express amino acid receptors *pctA*, *B* and *C* (PAO1ΔpctABC) and in PAO1 incubated in Ringer containing a high concentration of mixed amino acids. Non-piliated PAKΔpilA swarmed normally towards wounded areas but bound infrequently to CFBE41o- cells. In contrast, both swarming and binding of PA to CFBE41o- cells near wounds were prevented in non-flagellated PAKΔfliC. Data are consistent with the idea that (i) PA use amino acid sensor-driven chemotaxis and flagella-driven swimming to swarm to CF airway epithelial cells near wounds and (ii) PA use pili to bind to epithelial cells near wounds.

## Introduction

*Pseudomonas aeruginosa* is an aerobic bacterium that inhabits a wide range of environmental niches, from soil to water to human hosts and can metabolize a wide range of nutrients. The ability to live in such a wide range of environments requires that the bacteria rapidly seek out nutrients using a combination of flagellar-driven swimming and chemotactic responses to chemical signals. *P*. *aeruginosa* is thought to have 26 different chemoreceptors (methyl-accepting chemotaxis proteins, MCPs), though the specific molecules these receptors sense have not all been identified [1]. Using both capillary tube and agar-growth methods, it has been discovered that different strains of *P*. *aeruginosa* respond to molecules expected to be present in wounds in animals, e.g., amino acids [2,3], peptides [4], inorganic phosphate [5], glucose [6], and Krebs cycle intermediates [7,8]. *P*. *aeruginosa* also responds to thiocyanates [9], and a variety of aromatic compounds, though the chemotactic responses can be either attractive or repellent depending on the receptor and signaling pathways [10,11]. When a bacterium’s MCP chemosensor binds its selective target, the MCP generates chemotactic signals that are communicated to the flagellum [1] *via* a series of chemotaxis intracellular signaling kinases (e.g., CheA and CheY), phosphatases (e.g., CheZ) and other cytosolic regulators and transfer proteins (e.g., CheW and CheB) [12–16] that form a phosphotransfer-relay. Ultimately this cascade leads to phosphorylation/dephosphorylation of a component of the flagellar motor (FliM) and change of its activity, either halting rotation (tumbling) or rotating clockwise or counterclockwise. Changes in the concentration of the attractant (or repellent) thereby result in directed swimming towards (or away from) the highest concentration of the attractant (or repellent) [1].

*P*. *aeruginosa* is also an opportunistic bacterium that infects human patients with compromised immune systems or burns or with the genetic disease cystic fibrosis (CF). The bacterial and/or host mechanisms involved in the near universal infection of the lungs of CF patients with *P*. *aeruginosa* have not been identified, d Previous studies from this and other labs showed that *P*. *aeruginosa* binding to cultured airway epithelial cells showed that the bacteria bound equally to CF and CFTR-corrected airway epithelia [17] and that binding occurred more prominently to the basolateral surfaces of epithelia with high transepithelial resistance and to regions near tight junctions in epithelia with low transepithelial resistance [18,19]. In addition, results in a previous study [18] were consistent with the idea that *P*. *aeruginosa* bound prominently to dead cells along the edges of scratch-induced wounds in lung airway epithelia. Such enhanced binding could have resulted from preferential binding of randomly swimming bacteria to the dead epithelial cells or from the chemotaxis-directed swimming of bacteria to the dead cells followed by nonselective binding.

The original purpose of the present work was to test the role of bacterial chemotaxis in the initial binding of *P*. *aeruginosa* to cultured CF airway epithelia. The hypothesis was that live-cell imaging of airway epithelial cells exposed to GFP-expressing *P*. *aeruginosa* (PAO1-GFP) would identify cells that preferentially attracted the bacteria, and that these sites might be informative about later stages of biofilm formation in CF airway epithelial monolayers. Initial studies showed that PAO1-GFP interacted with and bound only infrequently to epithelial cells in confluent regions of the monolayers. However, these bacteria were clearly attracted to and occasionally became immobilized on cells that had been damaged during preparation of the coverglass-grown cells for observation in the imaging microscope. We therefore expanded the study to measure directed migration and binding of *P*. *aeruginosa* to CF airway epithelial cells that were scratch-wounded in the presence of the bacteria. As far as we can tell, this study is the first to test the role of bacterial chemotaxis to amino acids in directing *P*. *aeruginosa* migration towards and subsequent binding to wounds in epithelia. We chose CFBE41o- cells for these studies because previous work showed they form confluent, polarized monolayers when grown on filters, and CFTR-corrected CFBE41o- cells are also available [20] to test the role of CFTR expression in experimental protocols. Though epithelial cells grown on coverglasses may not polarize completely, we used this approach to facilitate microscope imaging experiments.

We also compared the migration of PAO1-GFP to wounds of CF vs CFTR-corrected CF airway epithelia to test the role of CFTR in binding *P*. *aeruginosa* and CF epithelia incubated with acidic (characteristic of CF) or alkaline pH (characteristic of nonCF) [21,22] to determine whether altered pH affected bacterial chemotaxis or binding.

We then tested the role of bacterial chemotaxis towards wounded epithelial cells by comparing responses of PAO1-GFP with PAO1 that had a *Che1* cluster-deletion (PAO1ΔcheYZABW, no expression of chemotaxis regulatory component genes *cheY*, *cheZ*, *cheA*, *cheB* and *cheW*) [1] and PAO1 missing amino acid-sensing receptor genes *pctA*, *pctB and pctC* (PAO1ΔpctABC) [1]. Complementary studies tested responses of PAO1-GFP incubated in media that contained high concentrations of amino acids, which we predicted would reduce *P*. *aeruginosa* chemotaxis towards the epithelial wounds by saturating bacterial receptors.

We finally tested whether *P*. *aeruginosa* flagella and pili were involved in migration towards and binding to wounds.

## Results

### PAO1-GFP migrate rapidly to dying cells in scratch-wounded airway epithelia

**Fig 1:**
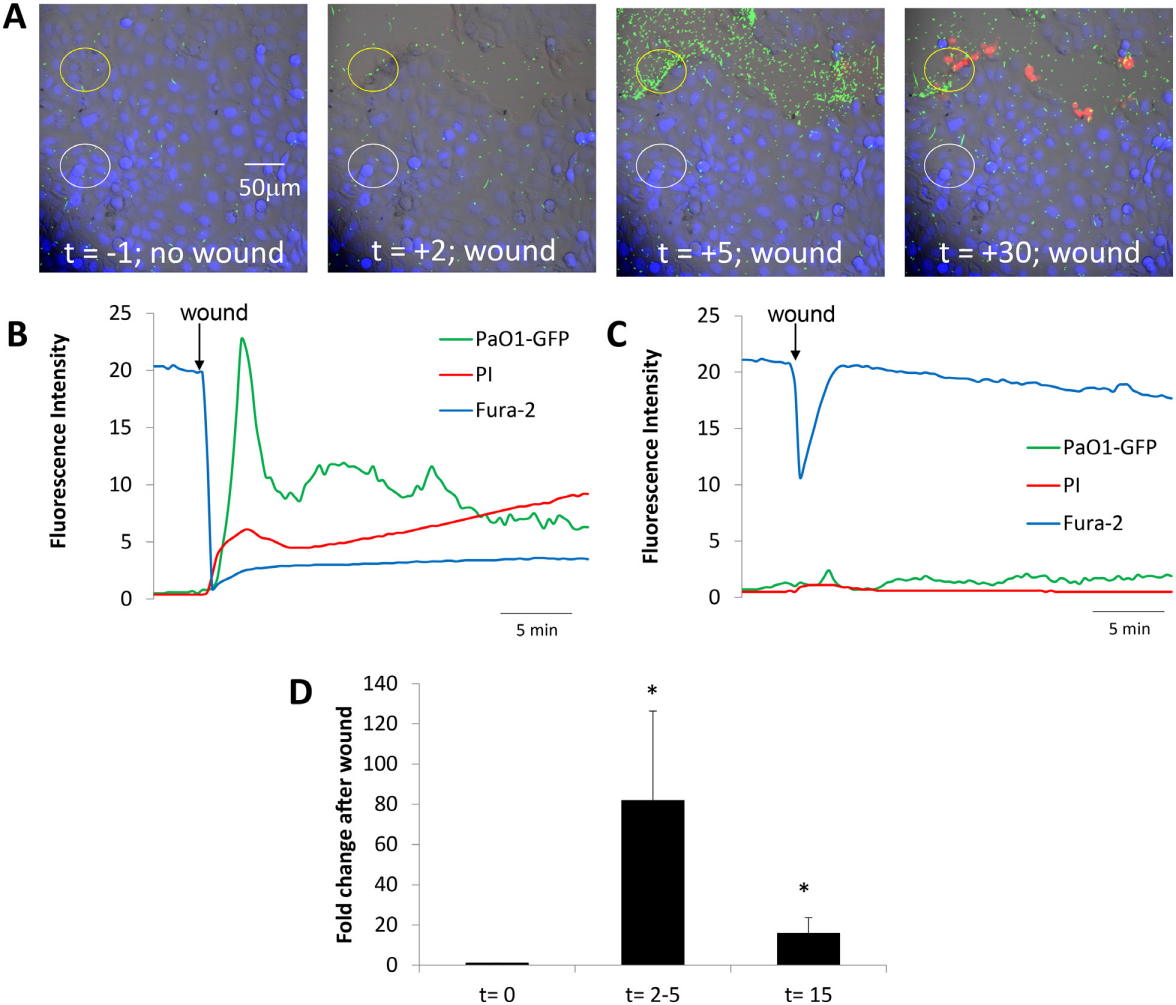
Time course of changes in CFBE41o- epithelia and PAO1-GFP following wounding. **A.** Images from S1 Movie showing overlays of DIC, fura-2 (blue), PI (red nuclei) and PAO1-GFP in control conditions (t = -1 min) and after wounding CFBE41o- cells (t = 2, 5 and 30 min). **B.** Quantitation of PAO1-GFP (green trace), live epithelial cells (blue) and dead epithelial cells (red) in the region of the wound shown by the yellow circle in A under control conditions and then following wounding (arrow). Fluorescence intensities were measured on background-subtracted images and expressed in arbitrary fluorescence units. **C.** Quantitation of PAO1-GFP (green trace), live epithelial cells (blue) and dead epithelial cells (red) in the adjacent non-wound region (white circle in A) under control conditions and then following nearby wounding (arrow). **D.** Average number of PAO1-GFP (normalized to bacteria in field before wounding) in the epithelial wounds (i.e., in regions shown by yellow circles in A) during control conditions (t = 0 min), at peak of bacterial migration to the wound (2–5 mins) and when bacteria returned to random swimming but some remained immobilized on both dead and live cells near the wound (t = 15 mins). Data are avg +/- SD (n = 5 experiments).

Results from a typical experiment in which a fura-2-loaded CFBE41o- airway epithelial cell monolayer was incubated in Ringer containing 1 μM PI and 5x10^6^ cfu/ml PAO1-GFP (2 MOI) is shown in S1 Movie (Supporting Information) and in individual images from the movie at the times shown in Fig 1A. Quantitation of the relative numbers of PAO1-GFP, fura-2 in the cells and PI in the nuclei during the experiment are presented in the graph shown in Fig 1B. At the beginning of the experiment, before wounding, bacteria migrated randomly over the surface of the epithelium, and only a few PAO1-GFP were observed in the microscope field at any one time. This pattern of random bacterial swimming over the surface and little or no immobilization on the epithelial cells of the confluent monolayer was consistently observed in all experiments before wounding, in regions adjacent to a wound and also in nonwounded epithelia for at least 30 mins (n = 5 experiments, not shown).

Scratch-wounding the CFBE41o- cell layer with a needle in the field of view caused many PAO1-GFP to swim rapidly towards the wound (“swarming”), usually within 20 sec (images were collected every 20 sec) but always within 1 min (Fig 1A and 1B, green trace). Swarming of bacteria to the wound became maximal 2–5 mins after the rapid loss of cells in the wound (coincided with large loss of fura-2, blue fluorescence) and followed a time course similar to the uptake of PI by damaged cells immediately adjacent to the wound (Fig 1A and 1B, green and red traces). The increase in number of PAO1-GFP in the wound likely resulted from migration of bacteria from regions above the plane of focus and from adjacent, nonwounded regions of the epithelium. After the peak of bacterial swarming to the wound at 2–5 mins and over the course of the next 5–10 mins, bacterial accumulation decreased, and they finally swam over the wound at rates like those observed in the controls. Some PAO1-GFP remained immobilized on dead cells along the wound, on nearby intact cells that retained fura-2 and did not take up PI and also to the cell-free region of the glass exposed during wounding (Fig 1A and 1B, green). *P*. *aeruginosa* migrated similarly to small, round wounds (as opposed to large, linear wounds), showing that the type of wound was unimportant in the increased *P*. *aeruginosa* migration to the wound (data not shown).

There was no change in the number of PAO1-GFP or in the number of dead cells in the nonwounded region adjacent to the damaged cells (Fig 1C, red and green) over the course of the experiment. However, there was a rapid decrease-then-increase in fura-2 fluorescence (ex 405 nm) in the nonwounded region (Fig 1C, blue trace). This transient change in fura-2 signal likely resulted from the transient increase-then-decrease in Ca_cyto_ (fura-2 fluorescence during 405 nm excitation increases when Ca_cyto_ decreases and decreases when Ca_cyto_ increases) in nonwounded cells adjacent to the wounded region. Similar transient Ca_cyto_ responses have been observed by others in epithelial cells near to wounds, and this is caused by release of ATP from dead/dying cells and activation of purinergic Ca^2+^ signaling in the nearby cells [23,24]. The small, relatively slow increase in blue fluorescence observed immediately adjacent to the wound following the wounding may have resulted from slow decreases in Ca_cyto_ in cells around the wound that were occurring following the large increase that occurred during (and was obscured by) the wounding. These changes in Ca_cyto_ indicated that Ca^2+^ signaling in intact cells near wounds may have been activated by ATP released from damaged cells, but this aspect of epithelial function was not investigated further.

A summary of the transient swarming followed by immobilization of PAO1-GFP at the wound regions is shown in Fig 1D. The variability in the peak swarming (at 2–5 mins) and steady state immobilization (after 15 mins) of PAO1-GFP along the wound may have resulted from unavoidable differences in sizes of the wounds. Resolution of the images was insufficient to determine whether there was selective immobilization of bacteria on dead vs. intact cells vs. denuded regions near the wounds, but comparison of Fig 1A–1D showed that compared to nonwounded regions, wounding caused both transient swarming of PAO1-GFP to the wound during the first 2–5 mins following wounding and later (15 min) immobilization of bacteria to cells and denuded regions near the wounds. In control experiments continuous 30 min exposure of confluent non-wounded CFBE41o- cells to PAO1-GFP showed no swarming or binding of bacteria (data not shown).

**Fig 2:**
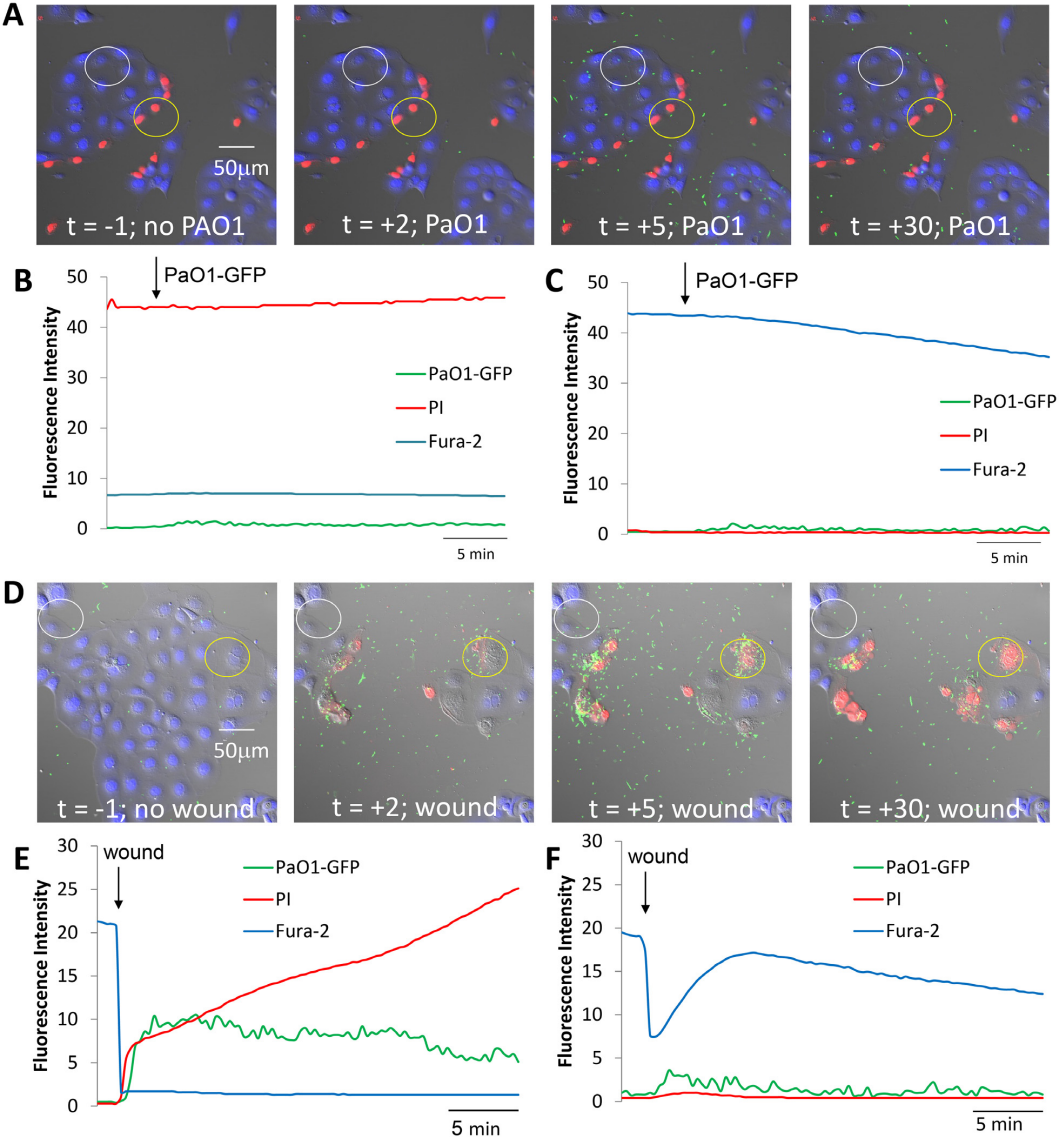
PAO1-GFP migration towards CFBE41o- cells that had been wounded and healed vs. freshly wounded. **A.** Images from S2 Movie showing a region of CFBE41o- cells incubated in Ringer containing PI that had been wounded and allowed to heal for 1 hr. Images were obtained before adding PAO1-GFP (t = -1 mins) and then after adding bacteria to the solution (t = 2, 5 and 30 mins). Typical of 3 similar experiments. **B.** Quantitation of PAO1-GFP (green trace), live epithelial cells (blue) and dead epithelial cells (red) in and near the wound (yellow circle in A) under control conditions and then following addition of PAO1-GFP (arrow). **C.** Quantitation of PAO1-GFP (green trace), live epithelial cells (blue) and dead epithelial cells (red) in an adjacent non-wounded region (white circle in A) under control conditions and then following wounding (arrow). **D.** Images from S3 Movie showing overlays of DIC, fura-2, PI and PAO1-GFP in control conditions (t = -1 min) and then after wounding CFBE41o- cells in the presence of bacteria (t = 2, 5 and 30 min). Typical of 3 similar experiments. **E.** Quantitation of PAO1-GFP (green trace), live epithelial cells (blue) and dead epithelial cells (red) in the wound region (yellow circle in D) under control conditions and then following wounding (arrow). **F.** Quantitation of PAO1-GFP (green trace), live epithelial cells (blue) and dead epithelial cells (red) in the adjacent non-wound region (white circle in D) under control conditions and then following wounding (arrow).

We next tested whether bacteria were attracted to dead or to dying epithelial cells by measuring PAO1-GFP migration to CFBE41o- epithelial cells that had been wounded and allowed to heal for one hr before being exposed to PAO1-GFP. Results from a typical experiment are shown in S2 Movie (Supporting Information) and summarized in Fig 2. PAO1 migrated randomly over the dead (red) cells along the wound (Fig 2A and 2B, green trace), over the cells in the adjacent nonwounded region, and also over the glass surface that surrounded the islands of epithelial cells (Fig 2A and 2C, green). After 30 mins there was no apparent specific accumulation of bacteria along the healed wound (nor anywhere else in the field of view). When this same coverslip was then moved to a new region and wounded again in the presence of PAO1-GFP, bacteria transiently swarmed to the new wound, and some bacteria remained immobilized on cells near the new wound (S3 Movie, Supporting Information; Fig 2D and 2E, green, blue and red). Nonwounded regions of the epithelium displayed the characteristic increase-then-decrease in fura-2 fluorescence, but there were only very small changes in bacterial accumulation and no changes in number of dead cells in nonwounded epithelial cells (Fig 2F, blue, green, red).

Data in S1–S3 Movies and Figs 1 and 2 showed that PAO1-GFP swarmed preferentially to dying, but not to dead, CF airway epithelial cells along wounds, and some bacteria remained immobilized on both live and dead cells and also to denuded regions near the wounds. The 5 and 30 min. time points shown in Fig 2A (and corresponding S2 Movie) also indicate that bacteria were not selectively attracted to the surface of the coverglass, as nonconfluent regions showed a similar distribution of bacteria compared to the wounded or confluent regions.

### *P*. *aeruginosa* swarm equally to wounds in CFBE41o- vs CFTR-corrected CFBE41o- cells

**Fig 3:**
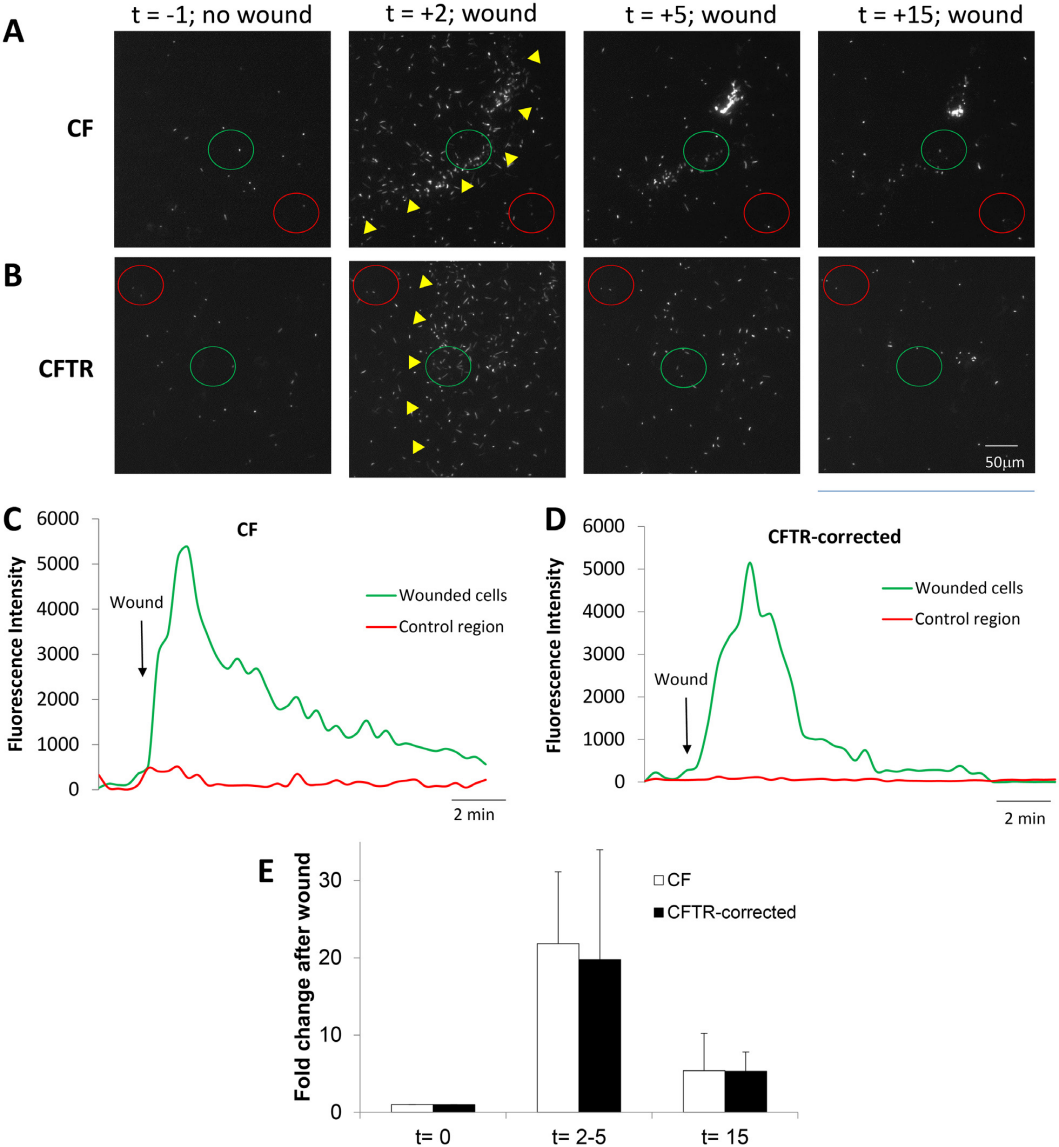
Similar responses of PAO1-GFP to epithelial wounds in CF vs. CFTR-corrected CFBE41o- cells. CFBE41o- and CFTR-corrected CFBE41o- cells were incubated in Ringer containing PAO1-GFP (2 MOI) and then wounded and imaged for 20 mins. **A.** Images from S4 Movie showing PAO1-GFP and unlabeled CFBE41o^-^ epithelial cells in control conditions (t = -1 min) and after wounding (yellow triangles) CFBE41o- cells (t = 2, 5 and 15 min). **B.** Images from S4 Movie showing PAO1-GFP and unlabeled CFTR-corrected CFBE41o^-^ epithelial cells in control conditions (t = -1 min) and after wounding (wound edge shown by yellow triangles) CFTR-corrected CFBE41o- cells (t = 2, 5 and 15 min). **C.** Quantitation of PAO1-GFP near the wounded CFBE41o^-^ cells (green circle in A) and non-wounded cells (red circle in A) under control conditions and then following wounding (arrow). **D.** Quantitation of PAO1-GFP near the wounded CFBE41o^-^ cells (green circle in B) and non-wounded cells (red circle in B) under control conditions and then following nearby wounding (arrow). **E.** Average number of PAO1-GFP (normalized to bacteria in field before wounding) in the CF and CFTR-corrected epithelial wounds (i.e., in regions shown by green circles in A and B) during control conditions (t = 0 mins), at peak of bacterial migration to the wound (2–5 mins) and after 15 mins. Data are avg +/- SD (n = 3 experiments).

The role of CFTR in the bacterial responses to epithelial wounding was tested by comparing swarming and immobilization of PAO1-GFP to cells and denuded regions near wounds in CFBE41o- (S4 Movie) and CFTR-corrected CFBE41o- cell (S5 Movie) monolayers. In control conditions, PAO1-GFP (2 MOI) migrated similarly in the Ringer solution above both CF and CFTR-corrected epithelial monolayers—there was minimal apparent immobilization of bacteria to the epithelial monolayers under control conditions. Wounding caused equivalent swarming (t = 2–5 mins) and apparent binding (t = 15 mins) of PAO1-GFP along the wounds (Fig 3A–3E).

**Fig 4:**
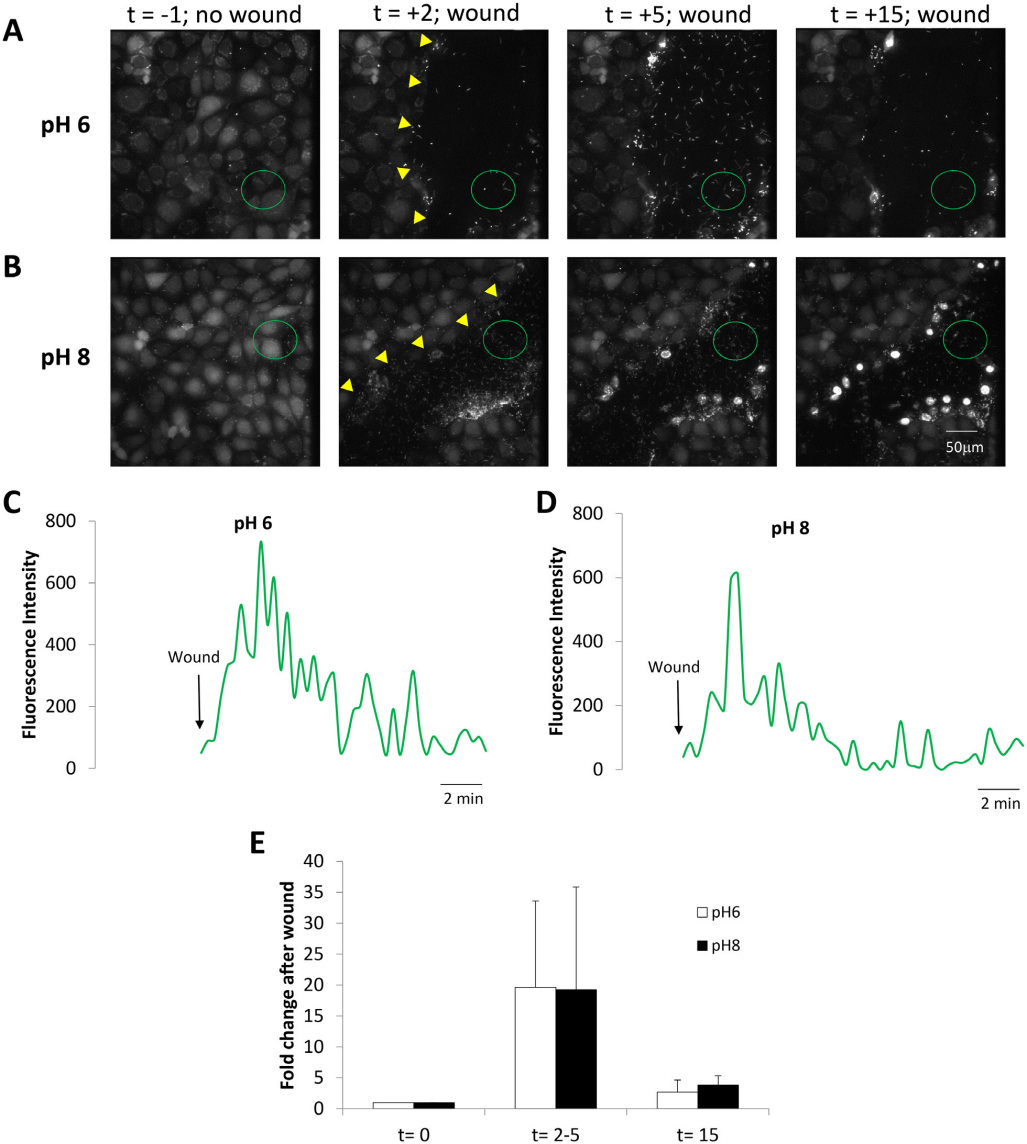
Similar responses of PAO1-GFP to wounds in CFBE41o- cells incubated in pH 8 vs pH 6 solutions. CFBE41o- cells (labeled with BCECF to for visualization) incubated in Ringer buffered to pH 6.0 or pH 8.0 and containing PAO1-GFP (2 MOI) were wounded and imaged. **A.** Images from S6 Movie showing PAO1-GFP and epithelial cells bathed in pH 6 Ringer in control conditions (t = -1 min) and after wounding (edge of wound marked by yellow triangles) epithelial cells (t = 2, 5 and 15 min). **B.** Images from S7 Movie showing PAO1-GFP and unlabeled epithelial cells bathed in pH 8 in control conditions (t = -1 min) and after wounding (edge of wound marked by yellow triangles) epithelial cells (t = 2, 5 and 15 min). **C.** Quantitation of PAO1-GFP near the wounded CFBE41o^-^ cells (green circle in A) bathed in pH 6 Ringer beginning when wound removed epithelial cells (arrow). **D.** Quantitation of PAO1-GFP near the wounded CFBE41o^-^ cells (green circle in B) bathed in pH 8 Ringer beginning when wound removed epithelial cells (arrow). **E.** Average number of PAO1-GFP (normalized to bacteria in field before wounding) in the epithelial wounds (i.e., in regions shown by green circles in A and B) during control conditions (t = 0 mins), at peak of bacterial migration to the wound (2–5 mins) and after 15 mins for the pH 6 and pH 8 experiments. Data are avg +/- SD (n = 4–5 experiments).

Bacterial responses to wounding were also compared for CFBE41o- cell monolayers (loaded with BCECF/AM to visualize the cells) incubated in Ringer buffered to pH 6.0 (S6 Movie) and pH 8.0 (S7 Movie). In control conditions before wounding, PAO1-GFP (2 MOI) migrated similarly above and with no apparent immobilization to CFBE41o- epithelial monolayers (S6 and S7 Movies). Wounding caused equivalent swarming of many bacteria to the wound region (t = 2–5 mins) and apparent binding of a few bacteria along the wound (t = 15 mins) (Fig 4A–4E). As summarized in Fig 4E, swarming and binding of PAO1-GFP to CFBE41o- wounds was unaffected by Ringer with pH 8 vs. pH 6.

### Bacterial mechanisms involved in migration and binding of *P*. *aeruginosa* to wounded CFBE41o- epithelial cells

**Fig 5:**
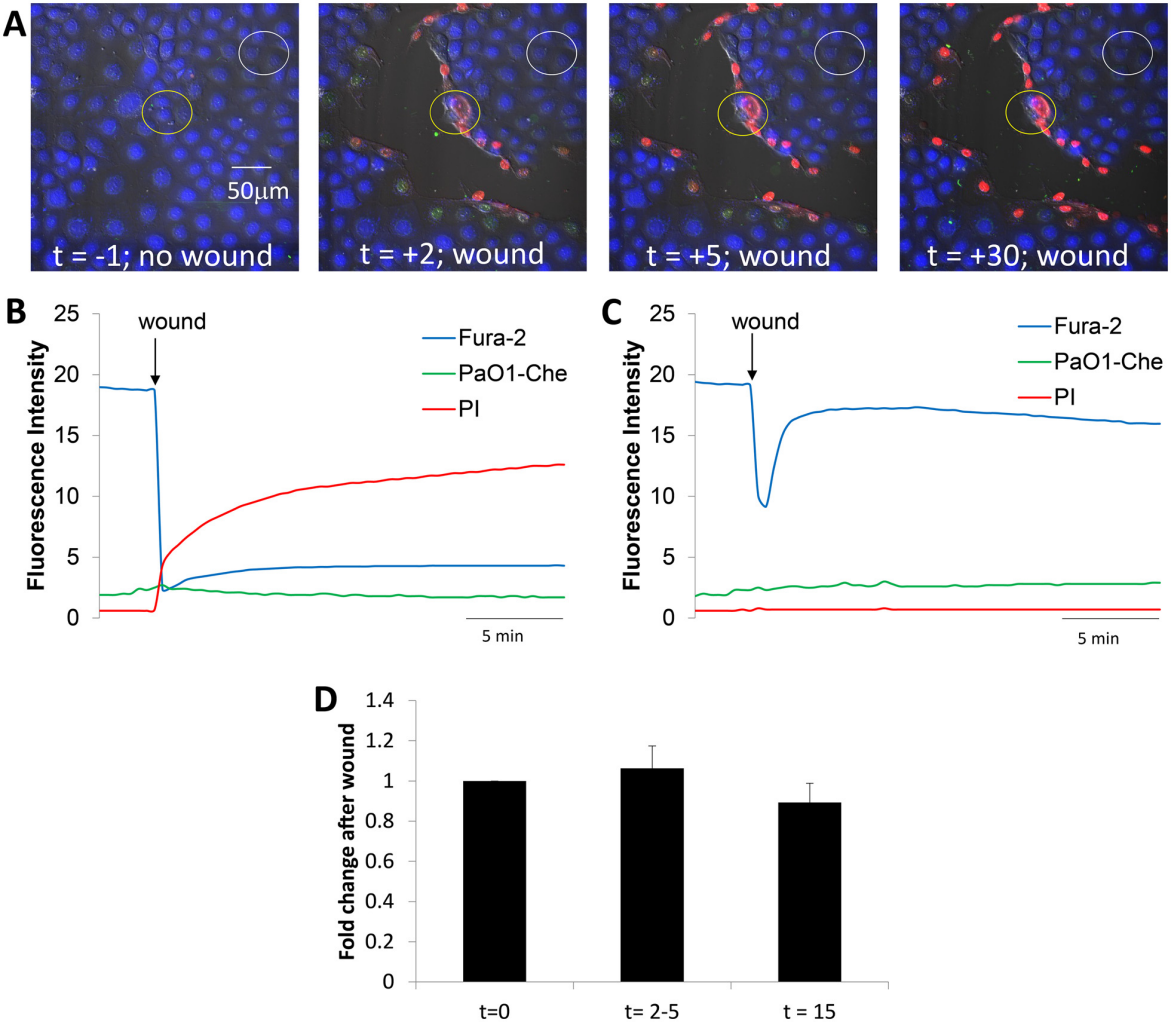
PAO1ΔcheYZBAW do not respond to CFBE41o- wounds. **A.** Images from S8 Movie showing CFBE41o- cells incubated in Ringer containing Syto 11-loaded PAO1ΔcheYZBAW (2 MOI) and PI before (t = -1 min) and at 2, 5 and 30 mins after wounding. **B.** Quantitation of PAO1ΔcheYZBAW (green trace), live epithelial cells (blue) and dead epithelial cells (red) near wound (yellow circle in A) under control conditions and then for 30 mins following wounding (arrow). **C.** Quantitation of PAO1-GFP (green trace), live epithelial cells (blue) and dead epithelial cells (red) in the adjacent non-wound region (white circle in A) under control conditions and then following wounding (arrow). **D.** Average number of PAO1ΔcheYZBAW (normalized to bacteria in field before wounding) at t = 0 mins (before the wound) and at 2–5 mins and 15 mins after wounding. Data are avg +/- SD, n = 4 experiments.

Experiments similar to those in Figs 1–4 were performed using PAO1 strains that had mutations in genes controlling chemotaxis signaling, amino acid-sensing and motility. We also tested whether high concentrations of amino acids in the Ringer would alter wound-directed swimming of PAO1. Syto11-labelled PAO1ΔcheYZBAW (2 MOI, missing cellular regulators of chemotaxis *cheY*, *cheZ*, *cheB*, *cheA* and *cheW*) moved randomly above the surface of control CFBE41o- monolayers under control conditions, and this pattern remained unchanged following scratch-wounding of the monolayer, even though the wound region of the epithelium showed typical loss of cells in the wound and uptake of PI into cells along the wound (S8 Movie; Fig 5A and 5B). The adjacent nonwounded portion of the CFBE41o- monolayer also showed typical decrease-then-increase in the fura-2 fluorescence (consistent with increase then decrease in Ca_cyto_) but little change in bacterial movements and no increase in dead epithelial cells (S8 Movie; Fig 5A and 5C). As summarized in Fig 5D, the number of PAO1ΔcheYZBAW in and around the wound remained constant before (t = 0) and then 2–5 mins and 15 mins following wounding.

**Fig 6:**
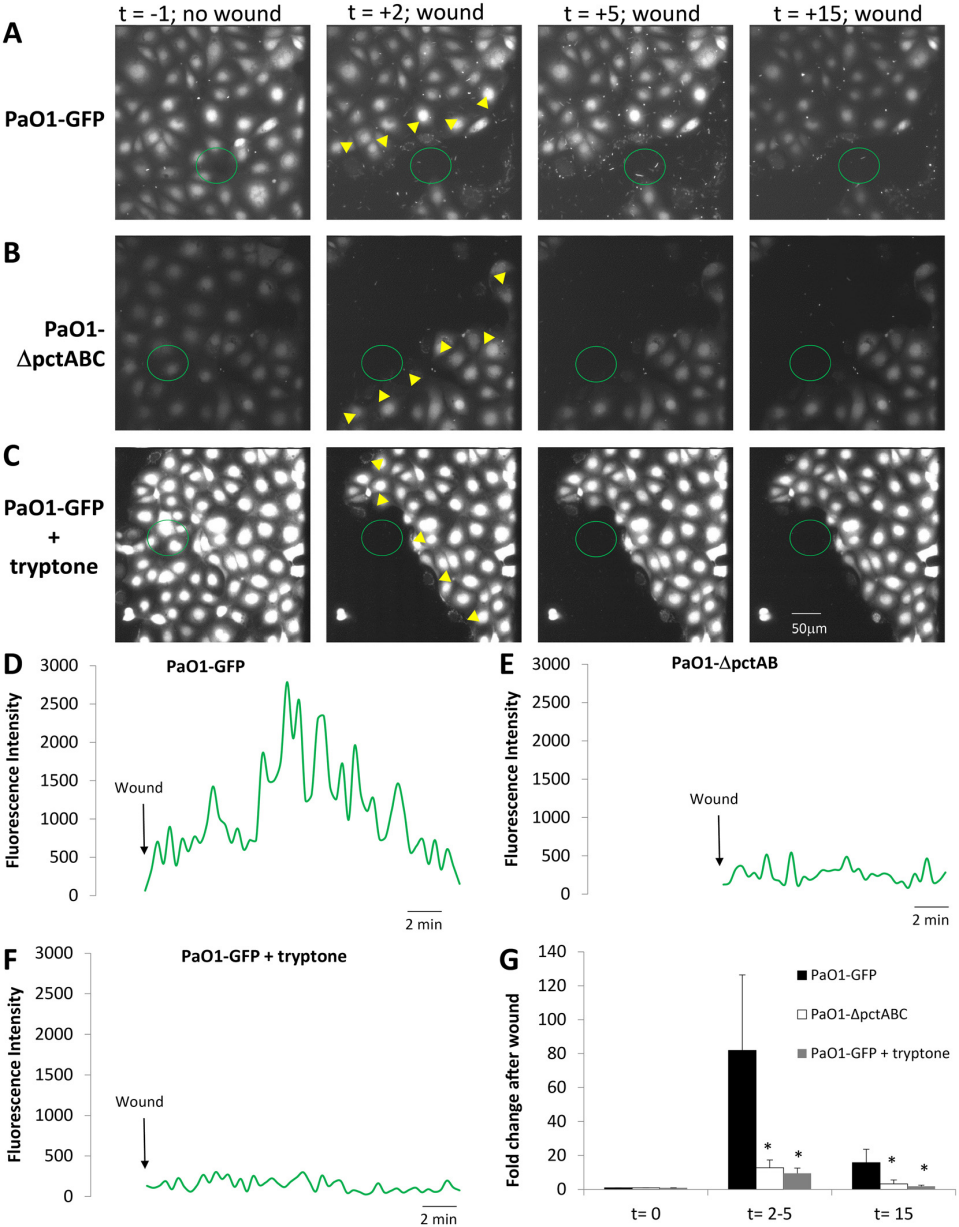
Role of leaked amino acids in swarming of PAO1 to wounded CFBE41o- cells. CFBE41o- cells (labeled with BCECF for visualization) were incubated in Ringer containing PAO1-GFP, with Syto 11-labelled PAO1ΔpctA,B,C or PAO1-GFP+tryptone (1% w/v) (2 MOI). **A.** Images from S9 Movie showing PAO1-GFP and epithelial cells in control conditions (t = -1 min) and after wounding (yellow triangles; t = 2, 5 and 15 min). **B.** Images from S10 Movie showing PAO1-ΔpctABC and epithelial cells in control conditions (t = -1 min) and after wounding (yellow triangles; t = 2, 5 and 15 min). **C.** Images from S11 Movie showing PAO1-GFP and epithelial cells with 1% tryptone in control conditions (t = -1 min) and after wounding (yellow triangles; t = 2, 5 and 15 min). **D.** Quantitation of PAO1-GFP near the wounded CFBE41o^-^ cells (green circle in A). **E.** Quantitation of PAO1-ΔpctABC near the wounded CFBE41o^-^ cells (green circle in B). **F.** Quantitation of PAO1-GFP near the wounded CFBE41o^-^ cells (green circle in C) in the presence of tryptone. **G.** Average numbers of bacteria (PAO1-GFP, PAO1ΔpctABC, and PAO1-GFP + tryptone) near the wound (in green circles, normalized to number before the wound) were measured before (t = 0) and after wounding (t = 2–5 and 15 mins). Avg +/- SD, n = 4–9 for each strain.

The role of amino acid-sensing receptors in bacterial swarming to dying epithelial cells was also tested by comparing PAO1-GFP (S9 Movie) and PAO1ΔpctABC mutants in amino acid sensing (S10 Movie) and by performing wounding experiments with PAO1-GFP in Ringer containing high concentrations of amino acids (tryptone) to compete with any wound-released amino acids that might cause wound-directed swimming of PAO1 (S11 Movie). As summarized in Fig 6A–6G (and compare S9–S11 Movies), swarming (t = 2–5 mins) and binding (t = 15 mins) responses exhibited by PAO1-GFP were greatly reduced in PAO1ΔpctABC (in-frame deletion of genes for the three amino acid receptors, *pctA*, *pctB* and *pctC*) [3,25] and also when the Ringer solution bathing the monolayers contained high concentrations of amino acids (1% w/v tryptone). None of the individual mutants (PAO1ΔpctA, PAO1ΔpctB or PAO1ΔpctC) showed significant reductions in swarming or immobilization near the wound to scratch-wounding of CFBE41o- monolayers compared to PAO1-GFP (data not shown). These data were consistent with the idea that rapid migration and binding of PAO1 to epithelial cells along wounds involved chemotaxis by the bacteria to multiple amino acids released from damaged cells.

**Fig 7:**
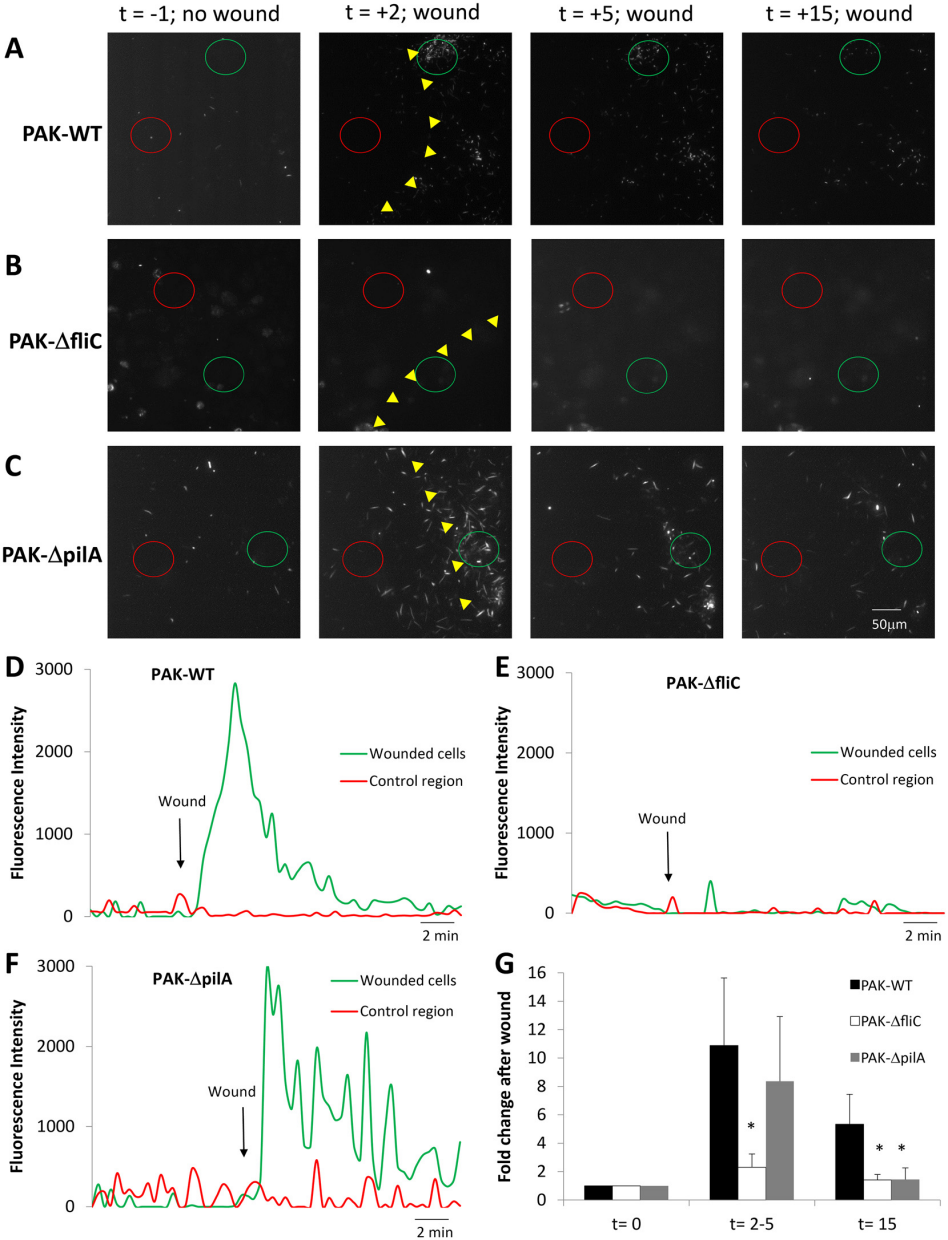
Altered chemotaxis of PAKΔfliC but not PAKΔpilA to wounds of CFBE41o- cell monolayers. CFBE41o- monolayers incubated in Ringer containing *P*. *aeruginosa* strains PAK-GFP, PAKΔflic and PAKΔpilA (2 MOI) were imaged under control conditions and following wounding. **A.** Images from S12 Movie showing PAK-GFP and epithelial cells in control conditions (t = -1 min) and after wounding (wound edge shown by yellow triangles; t = 2, 5 and 15 min). **B.** Images from S13 Movie showing PAK-ΔfliC and epithelial cells in control conditions (t = -1 min) and after wounding (wound edge shown by yellow triangles; t = 2, 5 and 15 min). **C.** Images from S14 Movie showing PAK-ΔpilA and epithelial cells in control conditions (t = -1 min) and after wounding (wound edge shown by yellow triangles; t = 2, 5 and 15 min). **D.** Quantitation of PAK-GFP near wounded CFBE41o^-^ cells (green circle in A) and in a control region (red circle in A). **E.** Quantitation of PAO1-ΔfliC near wounded CFBE41o^-^ cells (green circle in B) and in a control region (red circle in B). **F.** Quantitation of PAK-ΔpilA near wounded CFBE41o^-^ cells (green circle in C) and in a control region (red circle in C) in the presence of tryptone. **G.** Average numbers of bacteria (PAK-GFP, PAK-ΔfliC, and PAK-ΔpilA, normalized to number present before the wound) accumulating near scratch-wounded epithelia at times t = 0, 2–5 and 15 mins. Avg +/- SD, n = 3 for each strain.

The roles of flagella and pili in swarming to and immobilization near epithelial wounds were also tested by wounding CFBE41o- cell monolayers incubated in Ringer containing one of *P*. *aeruginosa* strains PAK-GFP, PAKΔfliC or PAKΔpilA. In control conditions before wounding, PAK-GFP behaved similarly to PAO1-GFP: bacteria migrated rapidly and randomly over the surface of the epithelium, and few PAK-GFP remained immobilized on the cells of the confluent monolayer (n = 3 experiments, not shown). Wounding the epithelium caused PAK-GFP to swarm and apparently bind to the cells around the wound (S12 Movie; Fig 7A, 7D and 7G), similar to that of PAO1-GFP (Fig 1). In contrast, PAKΔfliC (stained with Syto 11) moved slowly and randomly in the solution above the CFBE41o- monolayers (S13 Movie; Fig 7B, 7E and 7G) wounding had little effect on either swarming (t = 2–5 mins) or apparent immobilization of the bacteria along the wound (t = 15 mins). PAKΔpilA (stained with Syto 11) swam rapidly and randomly over the surface of the epithelium in control conditions, and they swarmed to wounds similar to PAK-GFP. However, there was reduced association of PAKΔpilA near epithelial wounds after 15 mins (S14 Movie, Supporting Information; Fig 7C, 7F and 7G).

**Fig 8:**
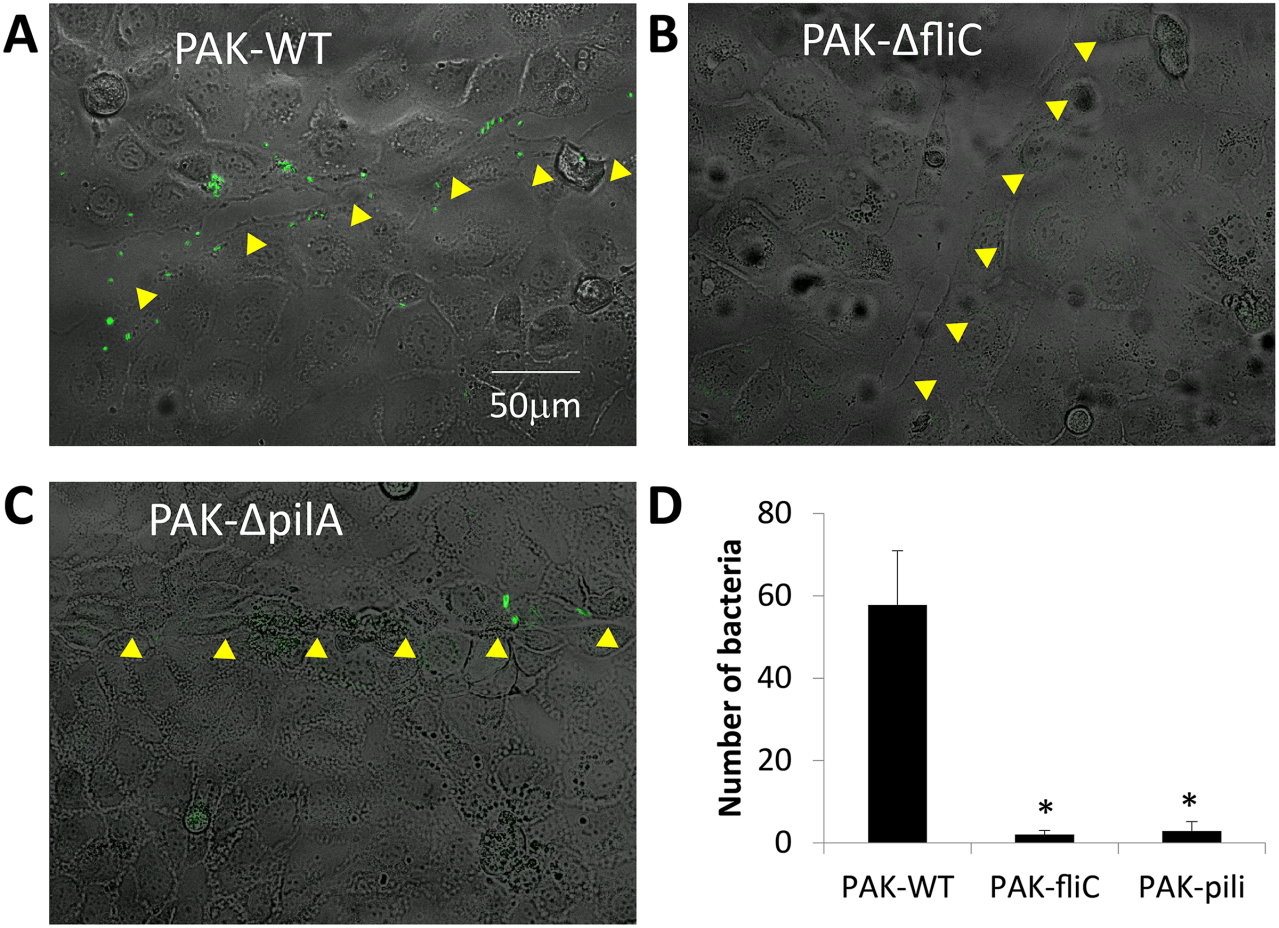
Flagella are required for swimming toward, and pili are required for binding of *P*. *aeruginosa* to wounded CFBE41o- cells. CFBE41o- monolayers were incubated in Ringer and wounded in the presence of *P*. *aeruginosa* strains PAK-GFP or Syto 11-labeled PAKΔfliC or Syto 11-labeled PAKΔpilA. After 30 mins, cells were rinsed and placed in the incubator for 2 hrs, then rinsed and imaged (DIC and green fluorescence). Yellow arrowheads show path of wound in healing CFBE41o- cells. **A.** PAK-GFP, **B.** PAKΔfliC, **C.** PAKΔpilA, **D.** Average (+/- SD, n = 3) number of bacteria bound along wounds of CFBE41o- cell monolayers.

A second set of experiments tested the roles of flagella and pili in PAK binding to cells along epithelial wounds. CFBE41o- cells were incubated for 30 min in Ringer containing either PAK-GFP or Syto 11-labeled PAKΔflic or Syto 11-labeled PAKΔpilA (2 MOI) and then wounded. This procedure was followed by rinsing the cells with Ringer to remove loosely bound bacteria and then returning CFBE41o- cells to the incubator for two hrs before rinsing them again and mounting them in the microscope to identify wounds and bound bacteria. Wounds were identified as regions of the epithelium where cells had been physically scraped away by the action of the needle leaving behind a denuded “scar” in the monolayer that had otherwise confluent regions. As shown for individual images of CFBE41o- cells, PAK-GFP bound to cells along the healing wounds (shown by arrow heads) (Fig 8A). In contrast, many fewer PAKΔfliC (Fig 8B) and PAKΔpilA (Fig 8C) bound along the wound. A summary of the average numbers of *P*. *aeruginosa* bound along wounds of CFBE41o- cells is shown in Fig 8D.

## Discussion

A major conclusion from this study was that *P*. *aeruginosa* use amino acid receptors pctA, pctB and pctC and che-dependent (cheYZABW) chemotaxis signaling to direct flagella-mediated swimming to airway epithelial cells around wounds. *P*. *aeruginosa* strains PAO1-GFP and PAK-GFP swarmed to wounds beginning within 20 sec following scraping of the epithelial cells and peaked at 2–5 mins. In contrast, *P*. *aeruginosa* exhibited random migration and minimal interaction with the apical surfaces of cells in control monolayers, in intact regions of monolayers that were near wounds and even in wounded epithelia that had been allowed to heal.

Bacterial swarming to the wound was, though, transient—the number of *P*. *aeruginosa* near the wound decreased markedly by 15 mins and remained low for the ensuing duration of experiments (20–35 mins). The few bacteria that remained in the wound appeared to become immobilized. Although swarming of *P*. *aeruginosa* to wounds was unaffected in single mutants PAO1ΔpctA (respond to glutamine but not to 20 other L-amino acids), PAO1ΔpctB (missing receptor for seven amino acids) and PAO1ΔpctC (missing receptor to glycine and glutamate) (data not shown), swarming was largely prevented in the triple mutant PAO1ΔpctABC. Consistent with these results, adding amino acids (as tryptone) to the solution bathing the epithelia reduced chemotaxis of PAO1-GFP to wounds to a similar extent as occurred in control Ringer with the triple mutant PAO1ΔpctABC. We also found very similar rates of chemotaxis of PAO1-GFP in Ringer containing 0 vs. 10 mM glucose or 0 vs. 1 mM ATP (Schwarzer, unpublished), indicating these potential attractants were likely not involved. *P*. *aeruginosa* swarming to CFBE41o- wounds was also unaffected by expressing CFTR or by solutions with pH 6–8.

A likely explanation for swarming of *P*. *aeruginosa* to epithelial wounds was that damaged cells around the wound that leaked fura-2 and took up PI also leaked cytosolic amino acids into the solution, and the bacteria used amino acid receptors and chemotaxis signaling to direct flagellar swimming to the wound. The localized, but transient nature of bacterial swarming to the wound likely resulted from the localized release of amino acids from dying cells followed by equilibration of the leaked cellular amino acids throughout the Ringer solution and loss of the localized high concentration of amino acids. Since *P*. *aeruginosa* swarm similarly to type III secretion-killed macrophages [26], swarming of *P*. *aeruginosa* to damaged, leaky cells may be common. Results from our experiments also provide verification of the roles of bacterial chemoreceptors and chemotaxis that have previously been studied using capillary tube and agar plate assays [1] and proposed to explain *S*. *typhimurium* swarming to damaged host cells [27].

The specificity of the swarming for amino acids described here is the first demonstration that *P*. *aeruginosa* use amino acid receptors and chemotaxis signaling for locating damaged airway epithelial cells. *Helicobacter pylori* use a similar chemotaxis-driven mechanism to locate urea-secreting epithelial cells in the stomach [28]. Although the CFBE41o- cells used for the present experiments (and for previous work on macrophages [26]) were grown on glass and complete polarization was not assured, there are several reasons for thinking that *P*. *aeruginosa* swarming to wounds observed here will occur similarly for both CF and nonCF airway epithelial cells grown *in vitro* on filters. (i) *P*. *aeruginosa* trigger similar inflammatory responses (NF- κB activation and IL8 secretion) in Calu-3 cells grown on filters and in plastic well plates [17,29]. (ii) *P*. *aeruginosa* binding to and type III secretion of exotoxins into Calu-3 airway epithelial cell monolayers occurs selectively at basolateral membranes and not at apical membranes in cells grown both as islands and on filters [18,30]. (iii) Activation of CFTR by forskolin and genistein in 16HBE41o- cells appears to occur similarly whether cells are grown on filters or on plastic in tissue culture well plates [31]. (iv) The release of cytosolic amino acids during physical wounding would be expected to occur similarly in polarized and nonpolarized epithelial cells. (v) Although some studies have reported altered inflammatory and apoptotic responses in CF vs nonCF or CFTR-corrected airway epithelia exposed to *P*. *aeruginosa* [32–37], *P*. *aeruginosa* swarming and immobilization on CFBE41o- cells was unaffected by CFTR expression and it seems unlikely that release of amino acids from wounded epithelial cells would be affected by the presence or absence of CFTR in the cells.

A second major conclusion from this study is that immobilization and binding of *P*. *aeruginosa* to airway epithelial cells near wounds also required amino acid receptors, chemotaxis signaling and flagella to locate the wound and pili (and perhaps flagella) to bind to cells, likely to (unidentified) sites that had been exposed by the wound. Importantly, immobilization of bacteria along wounds did not occur to wounds that had been allowed to heal for an hour before adding bacteria. The present work showing pilin-dependent binding to CFBE41o- cells was consistent with previous experiments showing pili-dependent binding of *P*. *aeruginosa* to basolateral sites on primary airway epithelia [38]. The present experiments extend previous work in showing that pili-dependent binding of *P*. *aeruginosa* to cells along the wounds increased dramatically if bacteria were present and able to swarm to damaged or dying cells during wounding. We propose that *P*. *aeruginosa* swarming to damaged epithelial cells brings the bacteria close to the wounds so that pili can bind to dead and live cells exposed during wounding.

A potential implication of the data in this paper is that injuries to airway epithelial cells (e.g., by toxic chemicals [39], cigarette smoke [40], particulates [41,42] or viral infections [43–45] may increase susceptibility of the airways to *P*. *aeruginosa* infections. Recent work has shown that epithelial wound healing may be different in cystic fibrosis patients [46,47], and the presence of *P*. *aeruginosa* in these patients could exacerbate the slow wound healing since bacteria would chemotax to and prevent healing of the wound. Such wounds could also provide nodes for formation of biofilms and ensuing downward spiral of bacterial binding and increased cell damage. Consistent with this idea, Garvis *et al* [48] showed that *P*. *aeruginosa* mutant *CheB2* (encodes a methylesterase involved in chemotaxis) exhibited attenuated virulence in both *C*. *elegans* and also in a mouse lung damage model. *P*. *aeruginosa* chemotaxis seems also to be important for the formation of biofilms [49].

## Materials and Methods

### Reagents

Syto 11, fura-2/AM, BCECF/AM and propidium iodide (all from Molecular Probes—Thermo Fisher Scientific) were dissolved in DMSO, DMSO/20% (w/v) Pluoronic (1:1) or water, respectively at 1 mM concentration. Tryptone was obtained from BD Biosciences. Other reagents and chemicals were obtained from Sigma (St. Louis, MO).

### Epithelial cells

The parent human bronchial CF (CFBE41o-, ΔF508/ΔF508) and CFTR-corrected (CFTR—CFBE41o-) cell lines (originally obtained from Dieter Gruenert, UCSF) were cultured as described previously [20,50] in either Dulbecco’s modified Eagle’s (DMEM) or Eagle's Minimum Essential (EMEM) media supplemented with 10% FBS, 2mM L-glutamine and 100 units/ml penicillin, and 100 μg/ml streptomycin. For imaging experiments, cells were passaged at a 1:2–1:5 dilution, and the remaining cell suspension was seeded directly onto round 18 mm coverglasses and grown in the bottoms of plastic well plates. Although epithelial cells may not polarize completely when grown on glass, we chose this approach to facilitate both bright field and fluorescence imaging microscopy. Cultures were maintained at 37°C in a humidified atmosphere of 5% CO_2_ and 95% air. As shown previously [20], both the parental CFBE41o- and the CFTR-corrected CFBE41o- cell lines maintained an epithelial phenotype and expressed Ca-activated Cl^-^ currents. The CFTR—CFBE41o- clone maintains a high level of transgene expression and forskolin-stimulated Cl^-^ secretion [20].

### Bacteria

*P*. *aeruginosa* strains PAO1 and PAK were used for these studies. PAO1 expressing GFP (PAO1-GFP) were originally obtained from George O’Toole (Dartmouth) [51]; PAK expressing GFP were generated as described previously [52]. Isogenic mutants of *P*. *aeruginosa* PAK lacking flagellin or pili (PAKΔfliC and PAKΔpilA) were engineered by in frame deletion of the *fliC* and *pilA* genes from the chromosome as described previously [52]. Isogenic mutants of PAO1 lacking genes for the amino acid-sensing receptors *pctA*, *pctB*, *pctC* and all three receptors (PAO1ΔpctA, PAO1ΔpctB, PAO1ΔpctC and PAO1ΔpctABC) and a *Che1* cluster-deletion mutant of PAO1 (PAO1ΔcheYZABW, no expression of chemotaxis regulatory components *cheY*, *cheZ*, *cheA*, *cheB* and *cheW*) were all obtained from Prof. Junichi Kato (Hiroshima University) and have been described previously [3].

All bacteria were grown overnight in Lysogeny broth medium at 37°C with vigorous aeration. Prior to experiments, bacteria were washed three times with PBS and were resuspended in Ringer's solution (145 mM NaCl, 2 mM KCl, 1.5 mM K_2_HPO_4_, 1 mM MgSO_4_, 10 mM HEPES, 2 mM CaCl_2_ and 10 mM glucose) at a concentration of 5x10^8^ cfu.ml^−1^ (OD_600_ = 0.5). PAO1 and PAK strains not expressing GFP were treated with 10 μM Syto 11 for 30 mins and then washed and resuspended in Ringer at a concentration of 5x10^8^ cfu.ml^−1^. Ringer solution containing Tryptone (1% w/v) was sterile filtered by passing the solution through a 0.2 μm Nalgene Syringe Filter (Thermo Scientific).

### Imaging methods

Epithelial cells were left untreated or were loaded with fura-2 by incubating CFBE41o- or CFTR-corrected CFBE41o- cells in Ringer at room temperature with 5 μM fura-2/AM, BCECF/AM, or no dye for 30 mins followed by rinsing and incubation for another 30 mins to recover from effects of dye loading. The cells were then mounted in the imaging chamber on the stage of an inverted confocal microscope (Nikon T2000 with spinning disk attachment or Zeiss LSM710) that also had DIC capabilities. 500 μl Ringer bathed the surface, and *P*. *aeruginosa* were added to the epithelial cells (5x10^6^ cfu/ml, 2 MOI), followed by 5–30 mins of no further treatments, depending on the experiment. Propidium iodide (1 μM) was included to identify dead epithelial cells as indicated in the figure legends. The imaging microscopes used for these experiments have been described previously [53,54].

Experiments were begun following the 5–15 min equilibration period by scraping the tip of a #20 gauge needle across the epithelial surface directly in the field of view using a 20x (0.75 NA) while recording images. Imaging of green *P*. *aeruginosa* (ex: 488 nm; em: 535 nm), blue fura-2 (ex: 405 nm; em: 506 nm) and red PI-stained nuclei (ex: 561 nm; em: 573–681 nm) was performed by focusing the 20x objective approximately 2–5 μm above the epithelial monolayer and collecting green, blue and red images sequentially every 20 secs for times indicated in the text. The 20x objective was chosen to facilitate rapid identification of the wound immediately following the scratch and also to provide a wide field of view for analysis of bacterial chemotaxis and binding to epithelial cells in a large swath of the wounds.

### Quantitation of bacterial accumulation along wounded and nonwounded regions of the epithelia

For experiments performed with the LSM 710 microscope, the Zen imaging software (Carl Zeiss) was used to outline and then quantitate blue (fura-2, to identify intact cells), red (PI, identify nuclei in dead cells) and green (*P*. *aeruginosa*, either GFP or Syto-labeled) fluorescence in background-subtracted images of regions along wounds and in nonwound/control regions of the epithelia during the 20–30 mins of the experiment. Wounded regions included cells that had been damaged (i.e., leaked fura-2 and accumulated PI in nuclei) and also intact cells (i.e., retained fura-2 and showed no PI in nuclei) and denuded regions (no fura-2 and no PI accumulation). Fluorescence intensities have been plotted on arbitrary scales to show the relative numbers of *P*. *aeruginosa* (green), intact epithelial cells (blue) and dead epithelial cells (red) during control conditions and then during and following wounding. A large decrease in blue (fura-2) fluorescence coincided with the damage or loss of cells that occurred during wounding provided a convenient “time” marker for the initiation of the in the imaging records.

For quantitation of bacteria during imaging with the Nikon T2000 spinning disk microscope green fluorescence intensities were recorded and analyzed with ImageJ software (NIH) using the threshold function to subtract background and the analyze particles function (size = 5^2^ pixel; circularity = 0.00–1.00) to count bacteria-specific fluorescence. The number of bacteria based on green fluorescence was normalized to bacteria recorded at the time of the wound and expressed as “fold change after wound”. Data in these experiments excluded regions of cells that had been stained with green BCECF. For Fig 8C individual cells were directly counted in the fields of view.

### Statistics

Quantitative data were presented as averages ± SD, where n = number of different biological samples or experiments. Statistical comparisons between individual treatment groups were done using t-tests for paired or unpaired samples as appropriate; p < 0.05 was considered statistically significant.

## Supporting Information

S1 Movie PAO1wt-GFP swim towards CFBE41o- wound. Fura-2-loaded CFBE41o- cells grown on a coverslip were incubated in Ringer containing PAO1-GFP (2 MOI) and PI while confocal and DIC images were recorded (1 image/20 sec). During control conditions bacteria swam randomly above the surfaces of the epithelial cells. After 5 mins of incubation the epithelium was wounded with a needle, which scraped away a swath of cells. In addition, cells adjacent to the wound leaked fura-2 and took up PI into nuclei (red). PAO1wt-GFP migrated rapidly to the wounded region, followed by a slow return to random migration over the surface during the ensuing 25 mins (total movie = 30 mins). At the end of the experiment, some bacteria remained immobile near wounded cells. Movie typical of >10 recordings.
(AVI)

S2 Movie PAO1wt-GFP swim randomly over CFBE41o- epithelia that have been wounded but allowed to heal for one hr. CFBE41o- cells were wounded, then allowed to heal for one hr in the incubator, followed by adding PAO1wt-GFP (2 MOI) to the wounded-then-healed cells. Results typical of n = 3 experiments.
(AVI)

S3 Movie PAO1wt-GFP swim towards fresh wound of CFBE41o- epithelia that had been wounded but allowed to heal for one hr. CFBE41o- cells from S2 Movie were moved to a new region for observation and scratch-wounding in the presence of PAO1-GFP. Results typical of n = 3 experiments.
(AVI)

S4 Movie PAO1-GFP swarm to wounded CF epithelial cells. CFBE41o- cells incubated in Ringer containing PAO1-GFP (2 MOI) were wounded after 2 mins. Results typical of n = 3 experiments.
(AVI)

S5 Movie PAO1-GFP swarm to wounded CFTR-corrected epithelial cells. CFBE41o- cells expressing CFTR and incubated in Ringer containing PAO1-GFP (2 MOI) were wounded after 2 mins. Results typical of n = 3 experiments.
(AVI)

S6 Movie PAO1-GFP swarm to wounded epithelial cells bathed in pH 6 Ringer. CFBE41o- cells (stained with BCECF/AM for visualization) were bathed in MES-buffered pH 6 Ringers containing PAO1-GFP (2 MOI) for 4 mins followed by wounding. Results typical of n = 5 experiments.
(AVI)

S7 Movie PAO1-GFP swarm to wounded epithelial cells bathed in pH 8 Ringer. CFBE41o- cells (stained with BCECF/AM for visualization) were bathed in HEPES-buffered pH 8 Ringers containing PAO1-GFP (2 MOI) for 3 mins followed by wounding. Results typical of n = 4 experiments.
(AVI)

S8 Movie PAO1ΔcheYZBAW are unaffected by epithelial wounding. CFBE41o- cells grown on a coverslip and loaded with fura-2 were incubated in Ringer solution containing Syto11-loaded PAO1Δ*cheYZBAW* (2 MOI) and PI while DIC and confocal images were recorded. Wounding the epithelium did not affect swimming of bacteria over the epithelial surface.
(AVI)

S9 Movie PAO1-GFP swarm to wounded epithelial cells. CFBE41o- cells (stained with BCECF/AM for visualization) bathed in Ringer containing PAO1-GFP(2 MOI) were wounded after about 2 mins of observation. Results typical of n = 9 experiments.
(AVI)

S10 Movie PAO1-ΔpctABC are unaffected by epithelial wounding. CFBE41o- cells (stained with BCECF/AM for visualization) were bathed in Ringer and wounded in the presence of PAO1-ΔpctABC stained with Syto 11 (2 MOI). Results typical of n = 4 experiments.
(AVI)

S11 Movie PAO1-GFP incubated in Ringer containing tryptone are unaffected by epithelial wounding. CFBE41o- cells (stained with BCECF/AM for visualization) bathed in Ringer containing tryptone (1%w/v) and PAO1-GFP (2 MOI) were wounded after 2 mins. Results typical of n = 3 experiments.
(AVI)

S12 Movie PAK-GFP swarm to wounded epithelial cells. CFBE41o- cells bathed in Ringer containing PAK-GFP (2 MOI) were wounded after 5 mins. Results typical of n = 3 experiments.
(AVI)

S13 Movie PAK-ΔfliC are unaffected by epithelial wounding. CFBE41o- cells bathed in Ringer containing PAK-ΔfliC (2 MOI) were wounded after 5 mins. Results typical of n = 3 experiments.
(AVI)

S14 Movie PAK-ΔpilA swarm to wounded epithelial cells. CFBE41o- cells bathed in Ringer containing PAK-ΔpilA (2 MOI) were wounded after 5 mins. Results typical of n = 3 experiments.
(AVI)

## References

[1] Kato J, Kim HE, Takiguchi N, Kuroda A, Ohtake H. *Pseudomonas aeruginosa* as a model microorganism for investigation of chemotactic behaviors in ecosystem. *J Biosci Bioeng*. 2008; 106: 1–7. 10.1263/jbb.106.1 18691523

[2] Kuroda A, Kumano T, Taguchi K, Nikata T, Kato J, Ohtake H. Molecular cloning and characterization of a chemotactic transducer gene in Pseudomonas aeruginosa. *J Bacteriol*. 1995; 177:7019–7025. 8522505 10.1128/jb.177.24.7019-7025.1995 PMC177577

[3] Taguchi K, Fukutomi H, Kuroda A, Kato J, Ohtake H. Genetic identification of chemotactic transducers for amino acids in Pseudomonas aeruginosa. *Microbiology* 1997; 143: 3223–3229. 9353923 10.1099/00221287-143-10-3223

[4] Kelly-Wintenberg K, Montie TC. Chemotaxis to oligopeptides by Pseudomonas aeruginosa. *Appl Environ Microbiol*. 1994; 60: 363–367. 8117090 10.1128/aem.60.1.363-367.1994 PMC201315

[5] Kato J, Ito A, Nikata T, Ohtake H. Phosphate taxis in Pseudomonas aeruginosa. *J Bacteriol*. 1992; 174:5149–5151. 1629173 10.1128/jb.174.15.5149-5151.1992 PMC206336

[6] Sly LM, Worobec EA, Perkins RE, Phibbs PV Jr. Reconstitution of glucose uptake and chemotaxis in Pseudomonas aeruginosa glucose transport defective mutants. *Can J Microbiol*. 1993; 39: 1079–1083. 8306210 10.1139/m93-163

[7] Alvarez-Ortega C, Harwood CS. Identification of a malate chemoreceptor in Pseudomonas aeruginosa by screening for chemotaxis defects in an energy taxis-deficient mutant. *Appl Environ Microbiol*. 2007;73: 7793–7795. 17933940 10.1128/AEM.01898-07 PMC2168054

[8] Lacal J, Alfonso C, Liu X, Parales RE, Morel B, Conejero-Lara F, et al Identification of a chemoreceptor for tricarboxylic acid cycle intermediates: differential chemotactic response towards receptor ligands. *J Biol Chem*. 2010;285: 23126–36. 10.1074/jbc.M110.110403 20498372 PMC2906306

[9] Ohga T, Masduki A, Kato J, Ohtake H. Chemotaxis away from thiocyanic and isothiocyanic esters in Pseudomonas aeruginosa. *FEMS Microbiol Lett*. 1993;113: 63–66. 8243984 10.1111/j.1574-6968.1993.tb06488.x

[10] Shitashiro M, Kato J, Fukumura T, Kuroda A, Ikeda T, Takiguchi N, et al Evaluation of bacterial aerotaxis for its potential use in detecting the toxicity of chemicals tomicroorganisms. *J Biotechnol*. 2003;101: 11–18. 12523965 10.1016/s0168-1656(02)00285-7

[11] Parales RE, Ditty JL, Harwood CS. Toluene-degrading bacteria are chemotactic towards the environmental pollutants benzene, toluene, and trichloroethylene. *Appl Environ Microbiol*. 2000;66: 4098–4104. 10966434 10.1128/aem.66.9.4098-4104.2000 PMC92264

[12] Falke JJ, Piasta KN. Architecture and signal transduction mechanism of the bacterial chemosensory array: progress, controversies, and challenges. *Curr Opin Struct Biol*. 2014; 29: 85–94. 10.1016/j.sbi.2014.10.001 25460272 PMC4268382

[13] Hazelbauer GL, Falke JJ, Parkinson JS. Bacterial chemoreceptors: High performance signaling in networked arrays. *Trends Biochem*. *Sci*. 2008; 33: 9–19. 10.1016/j.tibs.2007.09.014 18165013 PMC2890293

[14] Wuichet K., Zhulin I. B., Origins and diversification of a complex signal transduction system in prokaryotes. *Sci*. *Signal*. 2010; 3: ra50 10.1126/scisignal.2000724 20587806 PMC3401578

[15] Galperin M. Y.. A census of membrane-bound and intracellular signal transduction proteins in bacteria: Bacterial IQ, extroverts and introverts. *BMC Microbiol*. 2005; 5: 35 15955239 10.1186/1471-2180-5-35 PMC1183210

[16] Ulrich L. E., Koonin E. V., Zhulin I. B., One-component systems dominate signal transduction in prokaryotes. *Trends Microbiol*. 2005; 13: 52–56. 15680762 10.1016/j.tim.2004.12.006 PMC2756188

[17] Hybiske K, Fu Z, Schwarzer C, Tseng J, Do J, Huang N, et al Effects of cystic fibrosis transmembrane conductance regulator and DeltaF508CFTR on inflammatory response, ER stress, and Ca2+ of airway epithelia. *Am J Physiol Lung Cell Mol Physiol*. 2007; 293:L1250–60. 17827250 10.1152/ajplung.00231.2007

[18] Lee A, Chow D, Haus B, Tseng W, Evans D, Fleiszig S, et al Airway epithelial tight junctions and binding and cytotoxicity of Pseudomonas aeruginosa. *Am J Physiol*. 1999; 277: L204–17. 10409249 10.1152/ajplung.1999.277.1.L204

[19] Tran CS, Eran Y, Ruch TR, Bryant DM, Datta A, Brakeman P, et al Host cell polarity proteins participate in innate immunity to Pseudomonas aeruginosa infection. *Cell Host Microbe*. 2014; 15: 636–43. 10.1016/j.chom.2014.04.007 24832456 PMC4062193

[20] Illek B, Maurisse R, Wahler L, Kunzelmann K, Fischer H, Gruenert DC. Cl transport in complemented CF bronchial epithelial cells correlates with CFTR mRNA expression levels. *Cell Physiol Biochem*. 2008; 22: 57–68. 10.1159/000149783 18769032 PMC2927120

[21] Cho DY, Hwang PH, Illek B, Fischer H. Acid and base secretion in freshly excised nasal tissue from cystic fibrosis patients with ΔF508 mutation. *Int Forum Allergy Rhinol*. 2011; 1:123–7. 10.1002/alr.20028 22034590 PMC3199580

[22] Abou Alaiwa MH, Reznikov LR, Gansemer ND, Sheets KA, Horswill AR, Stoltz DA, et al pH modulates the activity and synergism of the airway surface liquid antimicrobials β-defensin-3 and LL-37. *Proc Natl Acad Sci U S A*. 2014; 111: 18703–8 10.1073/pnas.1422091112 25512526 PMC4284593

[23] Liu X, Wood PL, Parales JV, Parales RE. Chemotaxis to pyrimidines and identification of a cytosine chemoreceptor in *Pseudomonas putida*. *J Bacteriol*. 2009; 191: 2909–2916 10.1128/JB.01708-08 19251854 PMC2681813

[24] Sammak PJ, Hinman LE, Tran PO, Sjaastad MD, Machen TE. How do injured cells communicate with the surviving cell monolayer? *J Cell Sci*. 1997; 110: 465–75. 9067598 10.1242/jcs.110.4.465

[25] Glekas GD, Mulhern BJ, Kroc A, Duelfer KA, Lei V, Rao CV, et al The Bacillus subtilis chemoreceptor McpC senses multiple ligands using two discrete mechanisms. *J Biol Chem* 2012; 287: 39412–39418. 10.1074/jbc.M112.413518 23038252 PMC3501012

[26] Dacheux D, Goure J, Chabert J, Usson Y, Attree I. Pore-forming activity of type III system-secreted proteins leads to oncosis of *Pseudomonas aeruginosa*-infected macrophages. *Mol Microbiol*. 2001; 40:76–85. 11298277 10.1046/j.1365-2958.2001.02368.x

[27] Uhlman DL, and Jones GW. Chemotaxis as a factor in interactions between HeLa cells and *Salmonella typhimurium*. *J Gen Microbiol* 1982; 128: 415–418. 7042904 10.1099/00221287-128-2-415

[28] Huang JY, Sweeney EG, Sigal M, Zhang HC, Remington SJ, Cantrell MA, et al Chemodetection and Destruction of Host Urea Allows Helicobacter pylori to Locate the Epithelium. *Cell Host Microbe*. 2015; 18: 147–56. 10.1016/j.chom.2015.07.002 26269952 PMC4593702

[29] Pena J, Fu Z, Schwarzer C, Machen TE. Pseudomonas aeruginosa Inhibition of Flagellin-activated NF-kappaB and interleukin-8 by human airway epithelial cells. *Infect Immun*. 2009; 77: 2857–65. 10.1128/IAI.01355-08 19451246 PMC2708575

[30] Jacob T, Lee RJ, Engel JN, Machen TE. Modulation of cytosolic Ca(2+) concentration in airway epithelial cells by Pseudomonas aeruginosa. *Infect Immun*. 2002; 70:6399–408. 12379720 10.1128/IAI.70.11.6399-6408.2002 PMC130342

[31] Illek B, Lei D, Fischer H, Gruenert DC. Sensitivity of chloride efflux vs. transepithelial measurements in mixed CF and normal airway epithelial cell populations. *Cell Physiol Biochem*. 2010;26:983–90. 10.1159/000324011 21220929 PMC3221260

[32] Machen TE. Innate immune response in CF airway epithelia: hyperinflammatory? *Am J Physiol Cell Physiol*. 2006; 291: C218–30. 16825601 10.1152/ajpcell.00605.2005

[33] Hampton TH, Ballok AE, Bomberger JM, Rutkowski MR, Barnaby R, Coutermarsh B, et al Does the F508-CFTR mutation induce a proinflammatory response in human airway epithelial cells? *Am J Physiol Lung Cell Mol Physiol*. 2012; 303: L509–18. 10.1152/ajplung.00226.2011 22821996 PMC3468482

[34] Blohmke CJ, Mayer ML, Tang AC, Hirschfeld AF, Fjell CD, Sze MA, et al Atypical activation of the unfolded protein response in cystic fibrosis airway cells contributes to p38 MAPK-mediated innate immune responses. *J Immunol*. 2012; 189: 5467–75. 10.4049/jimmunol.1103661 23105139

[35] Losa D, Köhler T, Bellec J, Dudez T, Crespin S, Bacchetta M, et al Pseudomonas aeruginosa-induced apoptosis in airway epithelial cells is mediated by gap junctional communication in a JNK-dependent manner. *J Immunol*. 2014; 192:4804–12. 10.4049/jimmunol.1301294 24733844

[36] Oglesby IK, Chotirmall SH, McElvaney NG, Greene CM. Regulation of cystic fibrosis transmembrane conductance regulator by microRNA-145, -223, and -494 is altered in ΔF508 cystic fibrosis airway epithelium. *J Immunol*. 2013; 190: 3354–62. 10.4049/jimmunol.1202960 23436935

[37] Mayer ML, Sheridan JA, Blohmke CJ, Turvey SE, Hancock RE. The Pseudomonas aeruginosa autoinducer 3O-C12 homoserine lactone provokes hyperinflammatory responses from cystic fibrosis airway epithelial cells. *PLoS One*. 2011; 6:e16246 10.1371/journal.pone.0016246 21305014 PMC3031552

[38] Heiniger RW, Winther-Larsen HC, Pickles RJ, Koomey M, Wolfgang MC. Infection of human mucosal tissue by Pseudomonas aeruginosa requires sequential and mutually dependent virulence factors and a novel pilus-associated adhesin. *Cell Microbiol*. 2010; 12:1158–73. 10.1111/j.1462-5822.2010.01461.x 20331639 PMC2906647

[39] Cao X, Lin H, Muskhelishvili L, Latendresse J, Richter P, Heflich RH. Tight junction disruption by cadmium in an in vitro human airway tissue model. *Respir Res*. 2015; 16: 30 10.1186/s12931-015-0191-9 25851441 PMC4352288

[40] Rusznak C, Mills PR, Devalia JL, Sapsford RJ, Davies RJ, Lozewicz S. Effect of cigarette smoke on the permeability and IL-1beta and sICAM-1 release from cultured human bronchial epithelial cells of never-smokers, smokers, and patients with chronic obstructive pulmonary disease. *Am J Respir Cell Mol Biol*. 2000; 23: 530–6. 11017919 10.1165/ajrcmb.23.4.3959

[41] Psoter KJ, De Roos AJ, Mayer JD, Kaufman JD, Wakefield J, Rosenfeld M. Fine particulate matter exposure and initial Pseudomonas aeruginosa acquisition in cystic fibrosis. *Ann Am Thorac Soc*. 2015; 12: 385–91. 10.1513/AnnalsATS.201408-400OC 25594356

[42] Yoshizaki K, Brito JM, Toledo AC, Nakagawa NK, Piccin VS, Junqueira MS, et al Subchronic effects of nasally instilled diesel exhaust particulates on the nasal and airway epithelia in mice. *Inhal Toxicol*. 2010; 22: 610–7. 10.3109/08958371003621633 20429853

[43] Rezaee F, DeSando SA, Ivanov AI, Chapman TJ, Knowlden SA, Beck LA, et al Sustained protein kinase D activation mediates respiratory syncytial virus-induced airway barrier disruption. *J Virol*. 2013; 87: 11088–95. 10.1128/JVI.01573-13 23926335 PMC3807305

[44] Deng X, Yan Z, Luo Y, Xu J, Cheng F, Li Y, et al In vitro modeling of human bocavirus 1 infection of polarized primary human airwayepithelia. *J Virol*. 2013; 87: 4097–102. 10.1128/JVI.03132-12 23345515 PMC3624236

[45] Kong M, Maeng P, Hong J, Szczesniak R, Sorscher E, Sullender W, et al Respiratory syncytial virus infection disrupts monolayer integrity and function in cystic fibrosis airway cells. *Viruses*. 2013; 5:2260–71. 10.3390/v5092260 24056672 PMC3798900

[46] Schiller KR, Maniak PJ, O’Grady SM. Cystic fibrosis transmembrane conductance regulator is involved in airway epithelial wound repair. *Am J Physiol Cell Physiol* 2010; 299: C912–C921. 10.1152/ajpcell.00215.2010 20686068 PMC2980302

[47] Trinh NT, Bardou O, Privé A, Maillé E, Adam D, Lingée S, et al Improvement of defective cystic fibrosis airway epithelial wound repair after CFTR rescue. *Eur Respir J*. 2012; 40:1390–400. 10.1183/09031936.00221711 22496330

[48] Garvis S, Munder A, Ball G, de Bentzmann S, Wiehlmann L, Ewbank JJ, et al cheB2 in Pseudomonas aeruginosa virulence. *PLoS Pathog*. 2009; 5:e1000540 10.1371/journal.ppat.1000540 19662168 PMC2714965

[49] Schmidt J, Müsken M, Becker T, Magnowska Z, Bertinetti D, Möller S, et al The Pseudomonas aeruginosa chemotaxis methyltransferase CheR1 impacts on bacterial surface sampling. *PLoS One*. 2011; 6:e18184 10.1371/journal.pone.0018184 21445368 PMC3062574

[50] Schwarzer C, Fischer H, Kim EJ, Barber KJ, Mills AD, Kurth MJ, et al Oxidative stress caused by pyocyanin impairs CFTR Cl(-) transport in human bronchial epithelial cells. *Free Radic Biol Med*. 2008; 45:1653–62. 10.1016/j.freeradbiomed.2008.09.011 18845244 PMC2628806

[51] Bloemberg GV, O'Toole GA, Lugtenberg BJ, Kolter R. Green fluorescent protein as a marker for Pseudomonas spp. *Appl Environ Microbiol*. 1997; 63:4543–51. 9361441 10.1128/aem.63.11.4543-4551.1997 PMC168774

[52] Hybiske K, Ichikawa JK, Huang V, Lory SJ, Machen TE. Cystic fibrosis airway epithelial cell polarity and bacterial flagellin determine host response to *Pseudomonas aeruginosa*. *Cell Microbiol*. 2004; 6:49–63. 14678330 10.1046/j.1462-5822.2003.00342.x

[53] Schwarzer C, Ravishankar B, Patanwala M, Shuai S, Fu Z, Illek B, et al Thapsigargin blocks Pseudomonas aeruginosa homoserine lactone-induced apoptosis in airway epithelia. *Am J Physiol Cell Physiol*. 2014; 306:C844–55. 10.1152/ajpcell.00002.2014 24598360 PMC4010806

[54] Schwarzer C, Fu Z, Patanwala M, Hum L, Lopez-Guzman M, Illek B, et al Pseudomonas aeruginosa biofilm-associated homoserine lactone C12 rapidly activates apoptosis in airway epithelia. *Cell Microbiol*. 2012; 14:698–709. 10.1111/j.1462-5822.2012.01753.x 22233488 PMC4112999

